# Vaccine adjuvant activity of a TLR4-activating synthetic glycolipid by promoting autophagy

**DOI:** 10.1038/s41598-020-65422-1

**Published:** 2020-05-21

**Authors:** Yi-Ju Chou, Ching-Cheng Lin, Ivan Dzhagalov, Nien-Jung Chen, Chao-Hsiung Lin, Chun-Cheng Lin, Szu-Ting Chen, Kuo-Hsin Chen, Shu-Ling Fu

**Affiliations:** 1 Program in Molecular Medicine, School of Life Sciences, National Yang-Ming University and Academia Sinica, Taipei, 11221 Taiwan; 2 Institute of Microbiology and Immunology, National Yang-Ming University, Taipei, 11221 Taiwan; 3 Department of Life Sciences and Institute of Genome Sciences, National Yang-Ming University, Taipei, 11221 Taiwan; 4 Department of Chemistry, National Tsing Hua University, Hsinchu, 300 Taiwan; 5 Institute of Clinical Medicine, National Yang-Ming University, Taipei, 11221 Taiwan; 6 Department of Surgery, Far-Eastern Memorial Hospital, New Taipei City, 22060 Taiwan; 7 Institute of Traditional Medicine, National Yang-Ming University, Taipei, 11221 Taiwan

**Keywords:** Pharmacology, Drug development, Translational research, Molecular medicine, Adjuvants

## Abstract

Toll-like receptors (TLRs) play crucial roles in host immune defenses. Recently, TLR-mediated autophagy is reported to promote immune responses via increasing antigen processing and presentation in antigen presenting cells. The present study examined whether the synthetic TLR4 activator (CCL-34) could induce autophagy to promote innate and adaptive immunity. In addition, the potential of CCL-34 as an immune adjuvant *in vivo* was also investigated. Our data using RAW264.7 cells and bone marrow-derived macrophages showed that CCL-34 induced autophagy through a TLR4-NF-κB pathway. The autophagy-related molecules (Nrf2, p62 and Beclin 1) were activated in RAW264.7 cells and bone marrow-derived macrophages under CCL-34 treatment. CCL-34-stimulated macrophages exhibited significant antigen-processing activity and induced the proliferation of antigen-specific CD4+T cells as well as the production of activated T cell-related cytokines, IL-2 and IFN-γ. Furthermore, CCL-34 immunization in mice induced infiltration of monocytes in the peritoneal cavity and elevation of antigen-specific IgG in the serum. CCL-34 treatment *in vivo* did not cause toxicity based on serum biochemical profiles. Notably, the antigen-specific responses induced by CCL-34 were attenuated by the autophagy inhibitor, 3-methyladenine. In summary, we demonstrated CCL-34 can induce autophagy to promote antigen-specific immune responses and act as an efficient adjuvant.

## Introduction

Vaccine development holds a great contribution in infectious disease prevention and cancer immunotherapy. Vaccines contain both antigens and adjuvants to induce antigen (Ag)-specific antibody responses. Adjuvants are required to boost Ag-specific adaptive immune response in B cells and T cells. The current strategies of vaccine development to trigger early immune responses are via enhancing Ag uptake in antigen-presenting cells (APCs), providing appropriate microenvironments for APC activations, and further promoting the differentiation of naïve T cells into effector T cells^1,2^. Currently licensed adjuvants in clinical applications, such as AS04, MF59 and Alum, were reported to activate pattern recognition receptors (PRRs) and/or NLRP3 inflammasome, which trigger humoral antibody responses accompanied with T cell responses^1,3–5^. Recent studies showed that stimulation of autophagy is able to augment T cell responses via modulating the functions in both APCs and T cells^6^.

The autophagy/lysosome degradation pathway is an evolutionarily conserved stress response mechanism for survival^7^, and its dysregulation plays critical roles in human diseases^8^. During infection, one of the major immune mechanisms against pathogens is to induce canonical and noncanonical autophagy in macrophages. The canonical selective autophagy (xenophagy) selectively captures and degrades intracellular mycobacteria, such as *Mycobacterium tuberculosis* and *Listeria monocytogenes*^9,10^. Moreover, the noncanonical autophagy, LC3-associated phagocytosis (LAP), can accelerate pathogen clearance^11–15^. Furthermore, induction of autophagy increases the link between innate and adaptive immune responses by enhancing Ag processing in APCs^16^ as well as promoting the presentation of MHC class I and II Ag to CD8^+^ and CD4^+^ T cells^7,17–21^. Previous studies suggests that boosting autophagy by rapamycin, LPS or TLR-2-stimulating peptide can improve the vaccine efficacy of Bacillus Calmette-Guérin (BCG) against tuberculosis by promoting Ag presentation^22,23^. As mentioned above, autophagy regulates T cell responses via direct modulation of T cells and indirect modulation of APCs^6^. Therefore, autophagy-promoting agents may serve as potential vaccine/adjuvants candidates to enhance immune responses^24,25^.

Toll-like receptor 4 (TLR4) represents the first line of defense against pathogens and is an environmental autophagy sensor in innate immunity. TLR4-induced autophagy can overcome mycobacterial phagosome arrest and promote digestion of pathogens in autophagosomes^13^. It has been demonstrated that TLR4 agonists, such as aminoalkyl glucosaminide phosphates (AGPs), monophosphoryl lipid A (MPLA), and the MPLA-modified formulation (AS02 and AS04), can function as immune adjuvants in preclinical experiments and clinical applications^3,4,26^. Furthermore, TLR signal-stimulating particles can trigger LAP as a host defense mechanism^27^. Stimulation of TLR2 and TLR4 can enhance autophagy in APCs and promote immunogenicity of BCG vaccines against tuberculosis^22,23^. Overall, therapeutic intervention that modulates autophagy through TLR4 may serve as an effective strategy for vaccine or adjuvant development.

**Figure 1:**
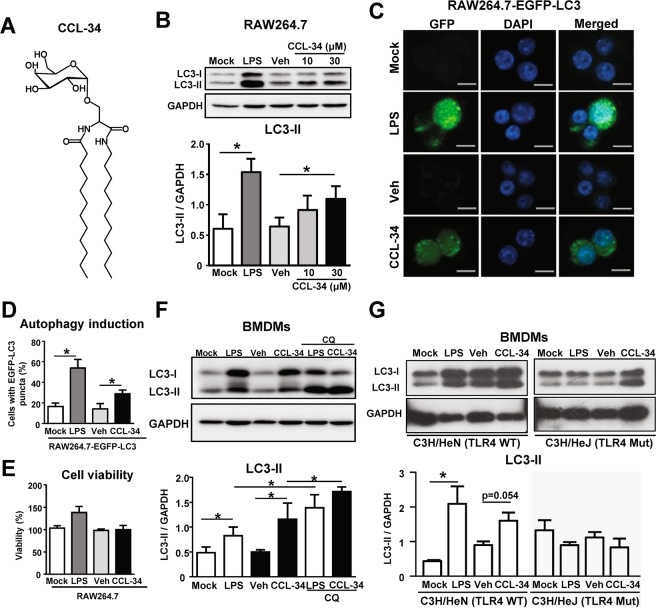
CCL-34 enhanced autophagy in macrophages in a TLR4-dependent manner. **(A)** The chemical structure of CCL-34 **(B)** CCL-34 promotes LC3-II production in macrophages. RAW264.7 cells were incubated with LPS (100 ng/mL), vehicle (0.1% DMSO), or CCL-34 (10 μM and 30 μM) for 24 hr. The LC3-II protein was detected by immunoblotting, using GAPDH as an internal control (n = 5). **(C,D)** CCL-34 promotes autophagosome formation in macrophages. RAW264.7-EGFP-LC3 cells were incubated with LPS (100 ng/mL), vehicle (0.1% DMSO), or CCL-34 (30 μM) respectively for 24 hr. Cells were fixed and stained with DAPI (blue). The quantification data (n = 5) are shown in **(D)**. **(E)** CCL-34 at 30 μM dose not cause cytotoxicity on RAW264.7 cells. RAW264.7 cells were treated with LPS (100 ng/mL), vehicle (0.1% DMSO), or CCL-34 (30 μM) for 24 hours, and the cell viability was measured by MTT assay (n = 3). **(F)** CCL-34 induces autophagy via enhancing autophagosome formation. Primary bone marrow-derived macrophages (BMDMs) were generated from C57BL/6 mice. BMDMs (1 × 10^6^) were incubated with LPS (100 ng/mL), vehicle (0.1% DMSO), or CCL-34 (30 μM) in the presence or absence of CQ (30 μM) for 24 hr. The LC3-II protein was detected by immunoblotting (n = 4). **(G)** CCL-34 induces autophagy in a TLR4-dependent manner. C3H/HeN (wild type) BMDMs and C3H/HeJ (TLR4-defective) BMDMs (1 × 10^6^) were incubated with LPS (100 ng/mL), vehicle (0.1% DMSO), or CCL-34 (30 μM) for 24 hr. The LC3-II protein was detected by immunoblotting (n = 3). All the data are shown as the mean ± SD, and *p < *0.05* indicates a significant difference *versus* the medium control or vehicle control analyzed using Student’s t-test, and the results were plotted using GraphPad Prism version 8.1.0 (www.graphpad.com). Total protein (50 μg for RAW 264.7 cells or 25 μg for BMDM) were analyzed using immunoblotting. The uncropped full-length blots of (**B**), (**F**) and (**G**) are shown in Supplementary Figure 3A–C.

Previously, our laboratory developed a novel synthetic glycolipid (designated as CCL-34; Fig. 1A) that induces the activation of macrophages and the maturation of dendritic cells in a TLR4-dependent manner. Additionally, CCL-34 exhibits anticancer activity *in vitro* and *in vivo* via TLR4-dependent activation of innate immunity^28–30^. Although the mRNA levels of T cell markers (CD4 and CD8) and related cytokines (IFN-γ and IL-12) have been observed to be increased in the tumor sites of CCL-34-treated mice^28^, whether CCL-34 can induce autophagy to facilitate Ag processing and thus enhance Ag-specific adaptive immunity remains unexplored. In this study, we demonstrate that CCL-34 can induce autophagy in APCs and facilitate antigen presentation to enhance T cell activation, as well as serve as an effective vaccine adjuvant.

## Results

### CCL-34 induced autophagy in macrophages through a TLR4-dependent pathway

We first investigated whether CCL-34, a TLR4 activator previously developed in our lab, could induce autophagy in macrophages. The conversion of LC3-I to LC3-II was measured to detect autophagy^31^. The LC3-II protein was significantly elevated under treatment with CCL-34 (30 μM) in RAW264.7 cells and bone marrow-derived macrophages (BMDMs) (Fig. 1B,F). To determine whether CCL-34 could promote autophagosome formation, the presence of EGFP-LC3 puncta was monitored in EGFP-LC3-expressing RAW264.7 cells (RAW264.7-EGFP-LC3) treated with CCL-34. The number of EGFP-LC3 puncta was significantly increased under treatment with CCL-34 (Fig. 1C,D). Additionally, CCL-34 treatment had no effect on the viability of RAW264.7 cells (Fig. 1E), indicating that CCL-34 did not induce autophagic cell death in macrophages. Subsequently, to investigate whether autophagic flux was also enhanced by CCL-34, LC3-II turnover under CCL-34 treatment was examined with or without cotreatment with chloroquine (CQ), an autophagy inhibitor that impairs autophagosome-lysosome fusion^32^. As shown in Fig. 1F, the CCL-34-treated BMDMs showed higher LC3-II levels under cotreatment with CQ, indicating that CCL-34 promotes autophagic flux in macrophages.

We next investigated whether CCL-34-induced autophagy is also TLR4-dependent. BMDMs were generated from either wild-type (C3H/HeN) or TLR4-defective (C3H/HeJ) mice and treated with CCL-34. As shown in Fig. 1G, the LC3-II protein was increased in the C3H/HeN BMDMs under CCL-34 treatment, whereas the LC3-II protein in CCL-34-treated C3H/HeJ BMDMs was not altered. In addition, the LC3-II protein level induced by CCL-34 in RAW264.7 cells was reduced upon co-treatment of TAK-242, a small-molecule inhibitor of TLR4 (Fig. S1A). Taken together, the results demonstrate that CCL-34 induces autophagy in a TLR4-dependent manner.

### Involvement of the Nrf2-p62 axis, Beclin 1 induction and NF-κB activity in CCL34-induced autophagy

**Figure 2:**
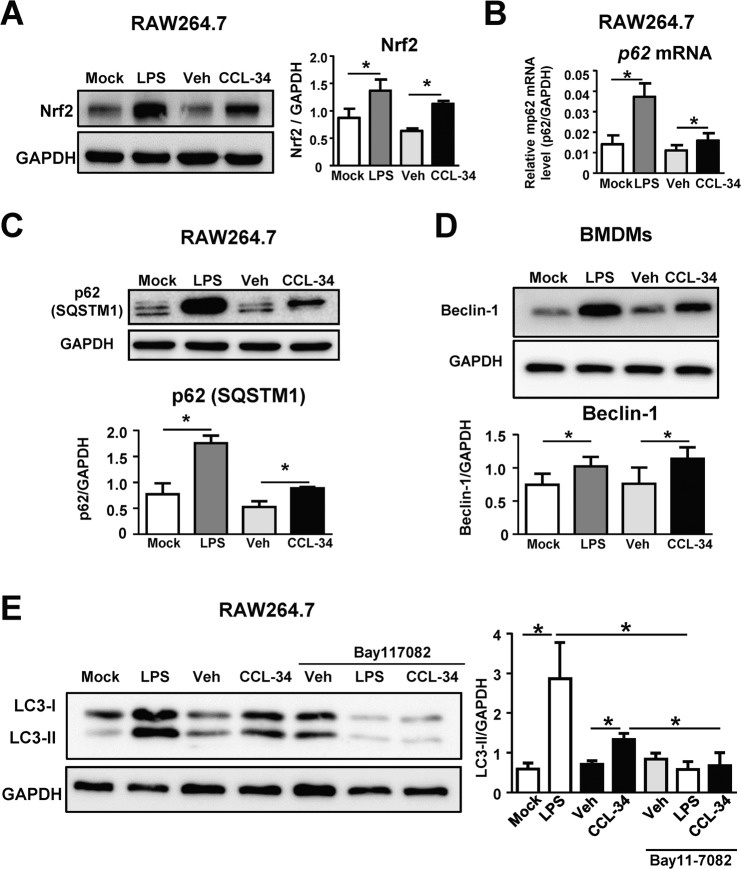
Involvement of the Nrf2-p62 axis, Beclin-1 induction and NF-κB activity in CCL34-induced autophagy. **(A**–**E)** RAW264.7 cells or BMDMs were incubated with LPS (100 ng/mL), vehicle (0.1% DMSO), or CCL-34 (30 μM). **(A,B)** RAW264.7 cells were treated with the candidate drugs for 12 hours. **(A)** CCL-34 induces Nrf2 protein expression. Nrf2 protein was detected by immunoblotting (n = 3), and **(B)** CCL-34 promotes p62 mRNA expression. p62 mRNA was detected by RT-qPCR (n = 6). **(C)** CCL-34 promotes p62 ptroten expression. RAW264.7 cells were treated with the candidate drugs for 24 hours and P62 protein was analyzed by immunoblotting (n = 3). **(D)** CCL-34 induces Beclin 1 protein expression. BMDMs generated from C57BL/6 were incubated with candidate drugs for 24 hours and the Beclin 1 protein was measured by immunoblotting (n = 5). **(E)** NF-κB activation is involved in CCL-34-mediated autophagy. RAW264.7 cells were treated with Bay11-7082 (10 μM) for 1 hour and then incubated with the candidate drugs for 24 hours. LC3-II protein was detected by immunoblotting (n = 3). The results were plotted using GraphPad Prism version 8.1.0 (www.graphpad.com). All the data are shown as the mean ± SD, and *p < *0.05* indicates a significant difference *versus* the medium control or vehicle control analyzed using Student’s t-test. Total protein (50 μg for RAW 264.7 cells or 25 μg for BMDM) were analyzed using immunoblotting. The uncropped full-length blots of (**A**), (**C**), (**D**), (**E**) are shown in Supplementary Figure 4A–D.

It is known that TLR4-mediated autophagy can be triggered by LPS^13^, and previous studies have shown that activation of the Nrf2-p62 axis is involved in TLR4-induced autophagy^33,34^. Nrf2 activation leads to transcriptional upregulation of p62, and the P62 protein can interact with LC3 to form an aggresome-like induced structure (ALIS), which promotes autophagosome formation. To investigate whether the Nrf2-p62 axis plays a role in CCL-34-induced autophagy, the expression of Nrf2 and p62 in CCL-34-treated RAW264.7 cells was measured using LPS as the positive control. The protein level of Nrf2 was elevated in CCL-34-treated RAW264.7 cells (Fig. 2A**)**, and both the mRNA and protein levels of p62 were significantly increased under CCL-34 treatment (Fig. 2B,C**)**. Furthermore, a key inducer of autophagy^35,36^, Beclin 1, was also increased under CCL-34 treatment in BMDMs **(**Fig. 2D**)**.

Previous studies have shown that constitutive activation of NF-κB is essential for TLR4-induced autophagy^37^. We next investigated whether NF-κB is involved in CCL-34-induced autophagy. When RAW264.7 cells were pretreated with the NF-κB inhibitor Bay11-7082, the CCL-34-induced elevation of LC3-II protein was abolished (Fig. 2E**)**. Together, these results suggest that the Nrf2-p62 axis, Beclin 1 and NF-κB are all involved in CCL-34-induced autophagy.

### CCL-34 induced the APC ability of macrophages and enhanced the Ag-specific T cell response

**Figure 3:**
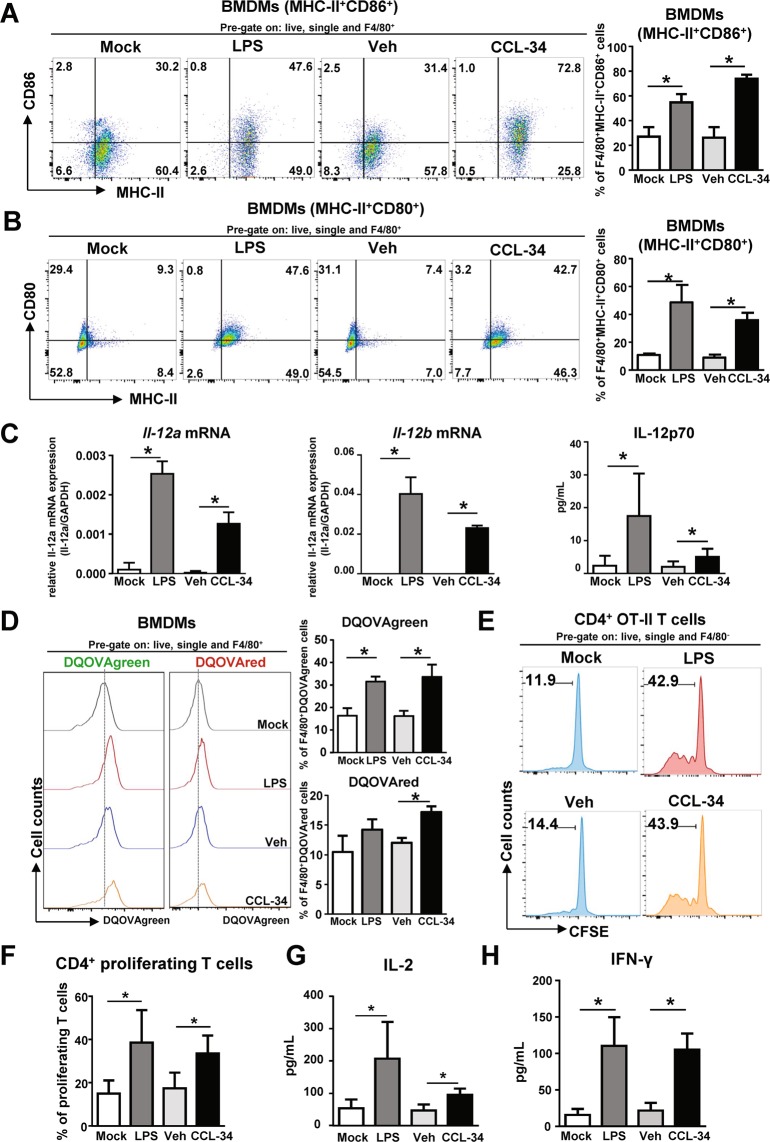
CCL-34-activated macrophages enhanced the proliferation and function of T cells *ex vivo*. BMDMs generated from C57BL/6 were incubated with LPS (100 ng/mL), vehicle (0.1% DMSO), or CCL-34 (30 μM) for 24 hours. **(A)** CCL-34 treatment increases CD86^+^MHC-II^+^F4/80^+^ cell population. The surface expression of CD86 and MHC-II on BMDMs (CD86^+^MHC-II^+^F4/80^+^ cells) is shown (n = 4). **(B)** CCL-34 treatment increases CD80^+^MHC-II^+^F4/80^+^ cell population. The surface expression of CD80 and MHC-II on BMDMs (CD80^+^MHC-II^+^F4/80^+^ cells) is shown (n = 3). **(C)** CCL-34 induced IL-12 in BMDM. *Il-12a (Left panel)* and *Il-12b (Middle panel)* mRNA was detected by RT-qPCR (n = 3). The production of IL-12p70 *(Right panel)* was measured using ELISA (n = 6) **(D)** CCL-34-activated macrophages show elevated Ag processing activity. BMDMs were incubated with candidate drugs in the combination with 5 μg DQ-OVA for 24 hours. *(Left panel)* A representative histogram showing the flowcytometric analysis of DQ-OVAgreen^+^ and DQ-OVAred^+^ BMDMs. *(Right panel)* The quantitative data are shown (n = 5). **(E,F)** CCL-34-activated macrophages enhance CD4^+^ T cell proliferation. Stimulated BMDMs and CFSE-labeled OT-II CD4^+^ T cells were cocultured for 5 days. The CFSE dilution, as an indicator of OT-II CD4^+^ T cell proliferation, was detected by flow cytometry. **(E)** A representative histogram; **(F)** The quantitative data (n = 4). **(G,H)** Co-culture of CCL-34-activated macrophages with CD4^+^ T cells increases the production of IL-2 and IFN-γ. The production of IL-2 **(G)** and IFN-γ **(H)** was measured using ELISA (n = 4). The results were plotted using GraphPad Prism version 8.1.0 (www.graphpad.com). All the data are shown as the mean ± SD, and *p < *0.05* indicates significant difference *versus* medium control or vehicle control analyzed using Student’s t-test. The gating strategies are presented in Supplementary Fig. 5A–D.

To address the immune-stimulating effect of CCL-34, we further investigated whether CCL-34 could promote Ag processing and presentation in APCs. The expression of MHC class II and the costimulatory signals (CD86 and CD80) in CCL-34-treated BMDMs were measured. As shown in Fig. 3A,B, the MHC-II^+^CD86^+^ and MHC-II^+^CD80^+^ cell populations were elevated in BMDMs under CCL-34 treatment. Furthermore, a critical cytokine produced by APCs during antigen presentation, IL-12, was elevated both in RNA and protein level in CCL-34-treated BMDMs (Fig. 3C). The Ag processing ability of CCL-34-treated macrophages was further determined using DQ ovalbumin (DQ-OVA) which is a self-quenched fluorescently labeled ovalbumin (OVA) and used as a model Ag. DQ-OVA emits green fluorescence (DQ-OVAgreen) upon proteolytic digestion, whereas red fluorescence (DQ-OVAred) is emitted when the digested fragments of DQ-OVA accumulate in organelles at high concentrations^38^. As shown in Fig. 3D, CCL-34-treated BMDMs exhibited a higher percentage of DQ-OVAgreen and DQ-OVAred fluorescence than the vehicle control, indicating that CCL-34-activated macrophages had an elevated Ag processing ability. Furthermore, CCL-34 treatment induced a higher percentage of DQ-OVAgreen^+^ RAW264.7 cells than vehicle group while the increased DQ-OVAgreen population was suppressed by the TLR4 inhibitor, TAK-242. This observation indicates that CCL-34 promotes Ag processing ability in a TLR4-dependent manner (Fig. S1B).

We further investigated whether the CCL-34-mediated increase in CD86 and CD80 levels and induction of Ag processing could lead to activation of Ag-specific T cells. OVA-specific CFSE-labeled CD4^+^ T cells were cocultured with BMDMs pretreated with OVA_323–339_ peptide alone or in combination with CCL-34, and the decrease in CFSE fluorescence was an indication of T cell proliferation. Only CCL-34 treatment of BMDMs resulted in the proliferation of OVA-specific CD4^+^ T cells and the production of activated T cell-related cytokines, IL-2 and IFN-γ (Fig. 3E–H). Taken together, these results demonstrate that CCL-34 can stimulate macrophages to activate Ag-specific T cell responses.

### Autophagy was involved in the CCL-34-mediated Ag presentation to T cells

**Figure 4:**
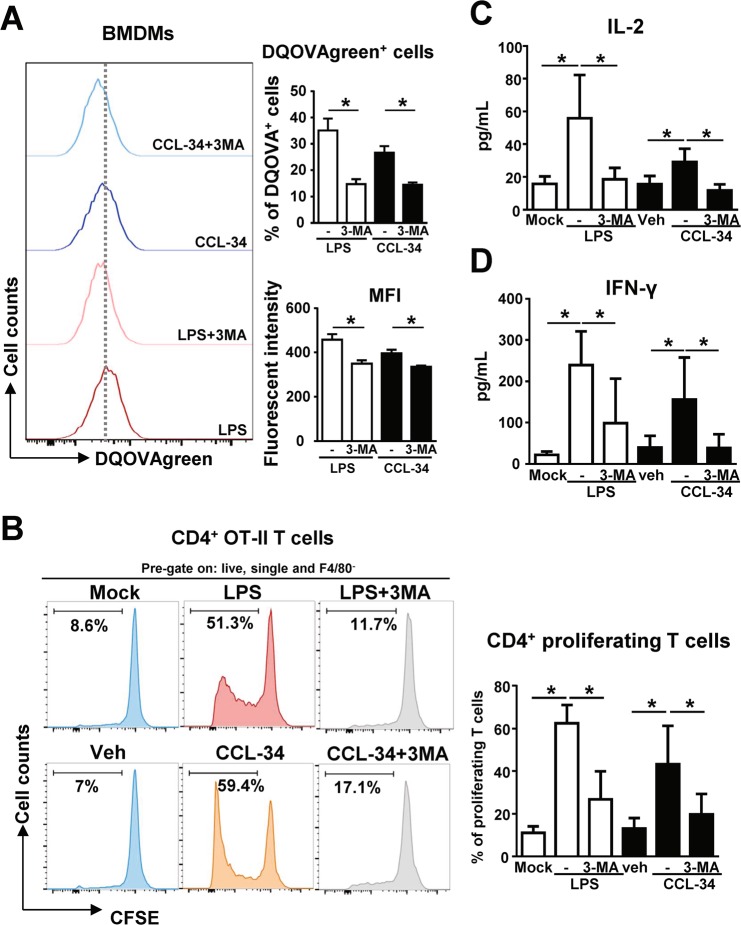
Autophagy is involved in the Ag-specific T cell responses induced by CCL-34-stimulated BMDMs. **(A)** The Ag processing activity of CCL-34-stimulate macrophage is suppressed by autophagy inhibitor 3-MA. BMDMs generated from C57BL/6 were treated with 3-MA (5 mM) for 1 hour and then incubated with LPS (100 ng/mL), vehicle (0.1% DMSO), or CCL-34 (30 μM) for 24 hours in the presence of 5 μg DQ-OVA (2.5 µg/mL) for 24 hours. *(Left panel)* Representative histogram showing flowcytometric analysis of DQ-OVA^+^ BMDMs; *(right panel)* the quantitative data (n = 3). **(B)** The CD4^+^ T cell proliferation stimulated by CCL-34-stimulate macrophage is blocked by 3-MA. BMDMs generated from C57BL/6 were stimulated with the candidate drugs in the combination with 5 μM OVA-peptide 323–339. The stimulated BMDMs and CFSE-labeled OT-II CD4^+^ T cells were cocultured for 5 days. A representative histogram and the quantitative data of multiple experiments are shown (n = 5). **(C,D)** The production of IL-2 and IFN-γ induced by co-culture of CCL-34 with CD4^+^ T cells is suppressed by 3-MA. The production of IL-2 (n = 6) **(C)** and IFN-γ (n = 7) **(D)** was measured using ELISA. The results were plotted using GraphPad Prism version 8.1.0 (www.graphpad.com). All the data are shown as the mean ± SD, and *p < *0.05* indicates significant difference *versus* the medium control or vehicle control analyzed using Student’s t-test. The gating strategies are presented in Supplementary Fig. 6A,B.

We next determined whether the CCL-34-induced enhancement of Ag processing and presentation by macrophages is regulated by autophagy. BMDMs were treated with CCL-34 in combination with or without the classical autophagy inhibitor, 3-MA, and analyzed for their Ag-presenting activity. As shown in Fig. 4A, DQ-OVAgreen fluorescence was decreased in CCL-34-stimulated BMDMs upon cotreatment with 3-MA, indicating that autophagy is involved in Ag processing in CCL-34-stimulated macrophages. Moreover, the increased proliferation of OVA-specific CD4^+^ T cells as well as the production of IL-2 and IFN-γ mediated by CCL-34-activated macrophages were also suppressed under treatment with 3-MA (Fig. 4B–D). These data indicate that autophagy plays a role in CCL-34-stimulated Ag processing and presentation to activate Ag-specific T cell responses.

### CCL-34 functions as an immune adjuvant *in vivo*

**Figure 5:**
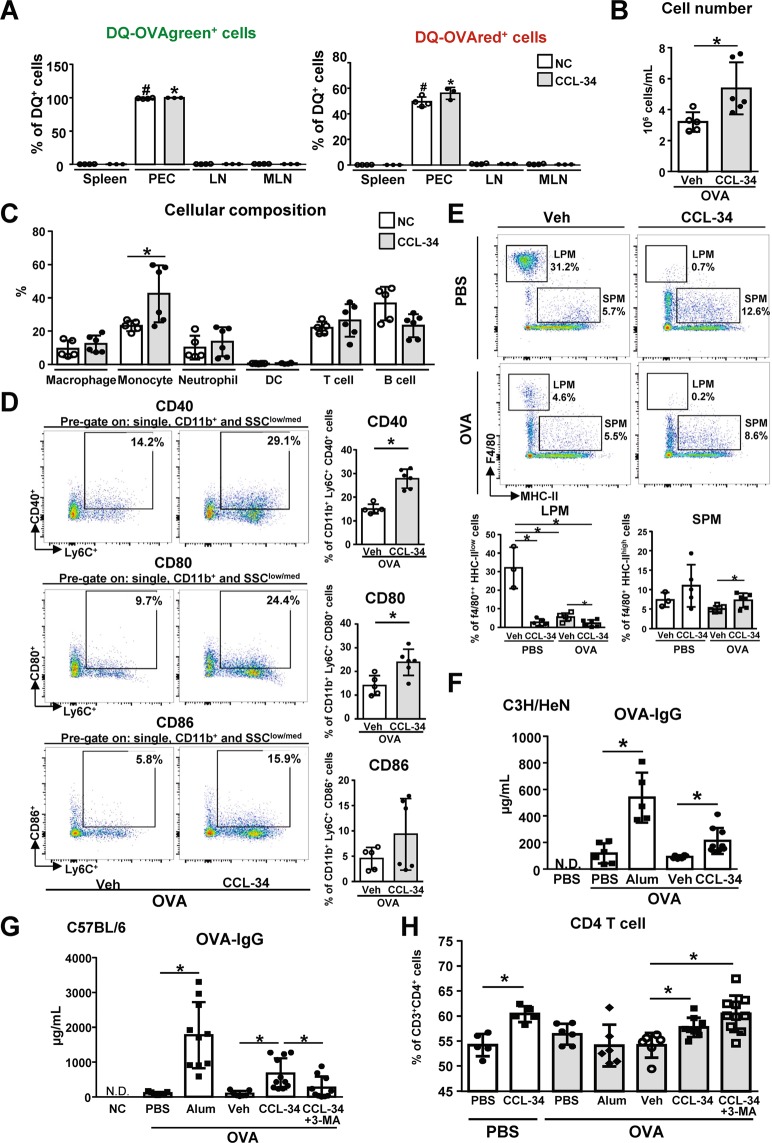
CCL-34 functions as an immune adjuvant *in vivo*. **(A)** The cells with Ag-processing ability were significantly increased in PEC of CCL-34-treated mice. C57BL/6 mice were injected intraperitoneally with DQ-OVA (1 mg/kg) in the presence or absence of CCL-34 (4 mg/kg). Cells were isolated from the spleen, peritoneal cavity, lymph nodes and MLN 24 hours later and analyzed by flow cytometry. The percentages of DQ-OVA green (digested OVA) and DQ-OVA red (digested and accumulated OVA) are shown. ^#^*p* < *0.05* and ^*^p < *0.05* indicate significant differences of PEC *versus* MLN, spleen or lymph nodes in vehicle group (n = 4) or CCL-34 group (n = 3). **(B**–**E)** C57BL/6 mice were injected intraperitoneally with OVA (100 μg/mouse) in the presence or absence of CCL-34 for 24 hours. **(B)** Total cell number of PEC in CCL-34/OVA-immunized mice (n = 6) is higher than control group (n = 5). Total cell number of cells isolated from the peritoneal cavity was measured. **(C)** The percentage of monocytes (SSC^low/med^CD11b^+^Ly6C^+^) in PEC is significantly increased in CCL-34/OVA-injected mice. The PECs were stained and analyzed by flow cytometry. The percentages of macrophages (F4/80^+^), monocytes (SSC^low/med^CD11b^+^Ly6C^+^), neutrophils (SSC^high^CD11b^+^Ly6C^+^), DCs (CD11c^+^MHC-II^high^), T cells (CD45^+^CD3^+^) and B cells (CD45^+^B220^+^) are shown. **(D)** The percentages of CD80^+^ and CD40^+^ monocytes in PEC are increased in CCL-34/OVA-injected mice. Representative scatter plots and the percentage of monocytes expressing CD40, CD80 or CD86 at 24 hours after injection are shown. **(E)** The percentage of large peritoneal macrophages is reduced while the percentage of small peritoneal macrophages is increased in PEC of CCL-34/OVA-injected mice. The PECs were stained and analyzed by flow cytometry. The percentages of large peritoneal macrophages (LPM, F4/80^++^ MHC-II^low^) and small peritoneal macrophages (SPM, F4/80^+^ MHC-II^high^) are shown. **(F)** CCL-34 induces OVA-specific IgG production in CCL-34/OVA-immunized C3H/HeN mice. C3H/HeN **(F)** or C57BL/6 mice **(G**,**H)** mice were immunized with OVA (100 μg/mouse) alone or OVA formulated with Alum (80 mg/kg), vehicle (10% DMSO) or CCL-34 (4 mg/kg) on days 0, 7 and 14. The serum was collected on day 21 and analyzed using ELISA. **(F,G)** The endpoint (day 21) concentration of OVA-IgG in the serum of immunized mice is shown (the number of mice: PBS = 5, PBS plus OVA = 6, Alum plus OVA = 5, vehicle plus OVA = 6, and CCL-34 plus OVA = 9 for total two trials). **(G)** CCL-34-induced OVA-specific IgG in CCL-34/OVA-immunized C57BL/6 mice is suppressed by 3-MA treatment. The autophagy inhibitor 3-MA (20 mg/kg) was administered intraperitoneally 30 minutes before immunization. The serum was collected on day 21 and analyzed using ELISA (the number of mice: PBS = 5, PBS plus OVA = 7, Alum plus OVA = 5, vehicle plus OVA = 7, CCL-34 plus OVA = 11, and CCL-34, 3-MA plus OVA = 10 for total two trials). **(H)** The percentage of CD3^+^CD4^+^ T cells is increased in CCL-34/OVA-immunized C57BL/6 mice. Cells isolated from the spleen of immunized mice described in (G) were stained and analyzed by flow cytometry. The percentage of CD4 T cells (CD3^+^CD4^+^) is shown (the number of mice: PBS = 5, CCL-34 = 5, PBS plus OVA = 7, Alum plus OVA = 5, vehicle plus OVA = 7, CCL-34 plus OVA = 11, and CCL-34, 3-MA plus OVA = 10 for total two trials). The results were plotted using GraphPad Prism version 8.1.0 (www.graphpad.com). All the data are shown as the mean ± SD, and *p < *0.05* indicates significant difference *versus* the control. The gating strategies are presented in Supplementary Fig. 7A–C.

We next investigated whether CCL-34 can serve as an adjuvant *in vivo*. C57BL/6 mice were immunized with CCL-34 and DQ-OVA via the intraperitoneal injections. We found that cells with the ability to process Ag (DQ-OVAgreen^+^ and DQ-OVAred^+^ cells) were significantly increased in peritoneal cavity cells (PECs) but not in spleen, mesenteric lymph nodes (MLN) or other lymph nodes at 24 hours after injection **(**Fig. 5A**)**. In addition, the total cell number of PECs in the CCL-34/OVA-injected mice was higher than in the OVA alone group **(**Fig. 5B**)**. Upon further phenotyping of PECs by flow cytometry, the percentage of monocytes (SSC^low/med^CD11b^+^Ly6C^+^) was significantly increased in CCL-34/OVA-injected mice **(**Fig. 5C**)**. Notably, the percentages of CD80^+^ and CD40^+^ monocytes were also higher in CCL-34/OVA-injected mice **(**Fig. 5D**)**. The percentage of large peritoneal macrophages (LPMs) was reduced following by the increased percentage of small peritoneal macrophages (SPMs) **(**Fig. 5E**)**. Overall, CCL-34 enhances recruitment and activation of innate immune cells, and promotes antigen processing *in vivo*.

**Figure 6:**
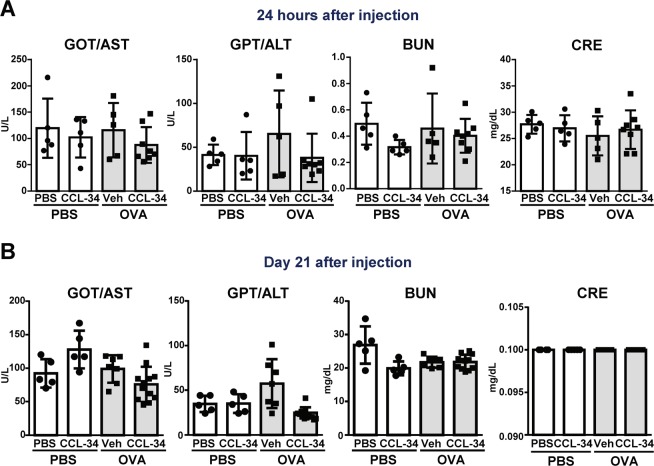
CCL-34 treatment did not cause cytotoxicity in C57BL/6 mice at early and late stages. **(A)** The toxicity index of CCL-34 at early stage. C57BL/6 mice were immunized with PBS or OVA (100 μg/mouse) formulated with vehicle (10% DMSO) or CCL-34 (4 mg/kg). The serum was collected 24 hours after injection, and then analyzed for the biochemical markers such as GOT, GPT, BUN, and CRE. **(B)** The toxicity index of CCL-34 at late stage. C57BL/6 mice were immunized with PBS or OVA (100 μg/mouse) formulated with vehicle (10% DMSO) or CCL-34 (4 mg/kg) on days 0, 7 and 14. The serum was collected on day 21 and then analyzed for the cytotoxic indexes such as GOT, GPT, BUN, and CRE. The results were plotted using GraphPad Prism version 8.1.0 (www.graphpad.com).

To further demonstrate that the activation of innate immunity induced by CCL-34 leads to activation of adaptive immune responses, the antibody responses in both C3H/HeN and C57BL/6 mice after immunization with CCL-34 and/or OVA were examined. The CCL-34/OVA-immunized mice showed higher levels of OVA-specific IgG in serum than the OVA-immunized group in both mouse strains, indicating that such immune responses were not strain-dependent **(**Fig. 5F,G**)**. The adjuvant activity of alum, the positive control, is notably higher than CCL-34 (Fig. 5F,G). In addition, the level of IgG1 and IgG2a, the important markers for T-helper 2 and T-helper 1 immune responses respectively, also showed the increasing tendency in CCL-34/OVA-immunized mice (Fig. S2A,B). The CD3^+^CD4^+^ T cells were also increased in the spleen of CCL-34/OVA-immunized mice **(**Fig. 5H**)**. Furthermore, to investigate whether autophagy participates in CCL-34-mediated immune responses, the mice were immunized with CCL-34 in combination with or without the autophagy inhibitor, 3-MA. As shown in Fig. 5G, the induction of OVA-specific IgG by CCL-34 was suppressed by 3-MA treatment. These data demonstrate that CCL-34 can function as an adjuvant *in vivo* by promoting autophagy. Additionally, CCL-34 did not show any toxicity based on the serum biochemistry data (Fig. 6).

## Discussion

Our previous studies found that the synthetic glycolipid, CCL-34, can activate macrophages and induce maturations of DCs in a TLR4-dependent manner^28–30^. CCL-34 also exhibits anticancer activity via macrophage-released NO-mediated *thoc1* downregulation in cancer cells^39^. In this study, we revealed that CCL-34 can induce TLR4-mediated autophagy and enhance Ag processing in macrophages. Furthermore, we also demonstrated CCL-34 could induce Ag-specific immune responses both *ex vivo* and *in vivo*.

Previous studies have shown that TLR4 is an environmental sensor of autophagy and that LPS-induced autophagy can overcome the mycobacterial phagosome block^13^. In addition, TLR4, TLR2 and TLR7 are known to promote autophagy in immune cells as a mechanism of pathogen elimination^24^. Several molecules and pathways have been reported to be involved in the regulation of TLR-mediated autophagy. TLR signaling increases the interaction of MyD88 and Trif with Beclin 1, leading to the induction of autophagy^40^. The autophagy-promoting molecule p62 (SQSTM1), which enhances the formation of ALIS and autophagosomes, is required for mycobactericidal activities^33,41^. The MyD88-NF-κB-DRAM1 axis is also critical for autophagic defenses against intracellular pathogens^37^. Our data in this study demonstrated the induction of Nrf2-p62 and Beclin 1 in CCL-34-activated macrophages (Fig. 2A–D). Furthermore, we also demonstrated that CCL-34-mediated autophagy was NF-κB-dependent (Fig. 2E). These data indicate that CCL-34-induced autophagy may play pivotal roles in TLR4-mediated mycobactericidal activities.

Vaccines play an important role in the prevention of infectious diseases by inducing pathogen-specific responses. One of the strategies for vaccine development is to combine recombinant antigens with adjuvants that modulate the immunogenicity of antigens as well as the microenvironments for activation of innate immunity^42^. To design safer and more effective vaccines, enhancing the immune effects of adjuvants via well-characterized mechanisms is necessary. In both preclinical studies and clinical trials, TLR4 agonists have well-defined mechanisms and have been proven to be potent immune stimulants to facilitate Ag delivery and generate appropriate microenvironments for the activation of APCs^43^. Therefore, TLR4 agonists are considered as potentially safe, universal and effective vaccine adjuvants for clinical applications^44–46^. Increasing autophagy-mediated Ag presentation has been demonstrated as a simple and powerful strategy to improve vaccine efficiency, as in the case of the BCG vaccine^22^. Autophagy-inducing small molecules or peptides have been demonstrated as potential adjuvants through their enhancement of Ag delivery, processing and presentation^47–49^. Combination with TLR2-stimulating peptide or LPS in BCG vaccines can induce autophagy in APCs and promote immunogenicity in mice^22,23^. Therefore, small molecules selectively triggering TLRs-mediated autophagy presumably increase immunogenicity and can potentially be developed as mycobacterial vaccines and adjuvants. In this study, we demonstrated that the TLR4 activator CCL-34 can induce autophagy, promote Ag-specific immune responses and act as an efficient adjuvant.

Our previous data have demonstrated that CCL-34 activates the TLR4 signaling pathway and induces cytokines related to the immune response^29,30^. In this study, we further showed that the Ag-specific T cells were activated **(**Fig. 3E–H**)** and the Ag-specific antibodies were elevated under CCL-34 treatment **(**Fig. 5F,G**)**. Based on our results and current studies of TLR4 agonists in other groups^50^, we suggested that CCL-34 may function as vaccine adjuvant for infectious diseases and cancers. For example, tumor antigens of breast cancers are considered as vaccine antigen in the presence of TLR4 agonists^50^, and CCL-34 can function as adjuvant for this type of cancer vaccine. Based on the current application of TLR4 agonists in infectious diseases^42^, L1 antigen of human papilloma virus and antigens (RTS, FMP012 or RH5.1) of *Plasmodium Falciparum* can be used with CCL-34. On the other hand, the adjuvant activity of CCL-34 in current study was investigated using the drug concentration, drug delivery method and immunization route based on our previous report^28^. Considering further improvement of adjuvant activity of CCL-34 *in vivo*, more effective immunization, more dosages and alternative immunization routes, such as the subcutaneous or intradermal injection^51^, may be tested in the future study. Moreover, the vaccine formulations can also play a critical role in vaccine efficiency via controlling the vaccine biodistribution and presentation to immune cells. The water-in-oil emulsion and liposomes are commonly used in TLR4-based adjuvant systems, such as AS01, AS02 and AS15^42,51^. Therefore, the water-in-oil emulsion and liposomes is suggested to develop potential formulation of CCL-34.

A previous study using i.p. injection as the immunization route showed that macrophages and neutrophils were the main cell population recruited into peritoneal cavity and then migrated into mesenteric lymph nodes (MLN)^52^. Our data also showed that macrophages and monocytes are the major innate immune cells recruited in peritoneal cavity of animal model (Fig. 5C). Therefore, we focused on the functions of macrophages in this study. LPMs (F4/80^high^MHC-II^low^) are the major macrophages that regulate the homeostasis of the peritoneal cavity and they migrate to the omentum during immune stimulation. SPMs (F4/80^low^MHC-II^high^) are the minor subset in the unstimulated peritoneal cavity but they become the major population for the secretion of cytokines and NO under immune stimulation or infection^53–58^. Previous studies showed that upon LPS stimulation, LPMs disappeared while the number of SPMs and monocytes increased in mice stimulated with LPS, TLR-4 agonists or Alum using i.p. injection^54,57,59,60^. LPS-stimulated LPMs may migrate into lymph nodes, serve as APCs, and trigger adaptive immune responses^54,57^. Furthermore, recent study found SPMs have the ability to present antigens to CD4^+^ T cells in MLNs^60^. In our study, we found SPMs were increased accompanied by the decrease of LPMs under CCL-34 immunization **(**Fig. 5E**)**, indicating that both LPMs and/or SPMs may function as the APCs in CCL-34-immunized mice. On the other hand, CCL-34 was shown to induce DC maturation *ex vivo* in our previous study^30^. The role of DCs in CCL-34-mediated adjuvant activity in animal model remains further investigation.

CD4^+^ T cells were also significantly increased in the spleens of CCL-34-immunized mice whereas the percentage of CD4^+^ T cells was not affected by 3-MA treatment **(**Fig. 5H**)**. Notably, the percentage of CD4^+^ T cells was increased under treatment with CCL-34 alone without antigens (Fig. 5H). Therefore, we hypothesized that CCL-34 may induce an antigen- or autophagy-independent increase of CD4^+^ T cells. In addition, although the percentage of CD4^+^ T cells was increased under treatment of CCL-34 and CCL-34 plus 3-MA, the functions of T cells *in vivo* were not examined in our study. Considering the critical role of autophagy in regulating T cell responses^6^, whether the functions of CCL-34-induced T cells are suppressed by 3-MA in vivo can be further explored in the future. Taken together, our data indicate that CCL-34 regulates both LPM and SPM functions and induces the percentage of CD4^+^ T cell which is similar to the immune stimulating response of LPS.

Although LPS is a natural adjuvant that activates TLR4 signaling pathways and has a profound effect on CD4^+^ T cell responses, its powerful adjuvant activity is associated with toxicity^61^. A high-dose LPS treatment can induce acute inflammation and lead to sepsis *in vivo*^62^. However, in the case of MPLA, an LPS-derived TLR4 agonist, toxicity and immunogenicity are not always linked^61,63^. CCL-34 is a synthetic TLR4 agonist that has a defined structure, and its structure is less complicated compared with that of other TLR4 agonists. Notably, our data showed that the dose used for an *in vivo* experiment in CCL-34-treated mice did not cause toxicity based on serum biochemical data, including GOT (glutamate oxaloacetate transaminase), GPT (glutamic pyruvate transaminase), BUN (*blood urea nitrogen)* and CRE (creatinine) measurements **(**Fig. 6**)**. In addition, activation of TLR4 by CCL-34 does not lead to high secretion of proinflammatory cytokines as LPS treatment dose^29^. Therefore, the immune response induced by CCL-34 may be safer than that induced by LPS.

Together, our results demonstrated that CCL-34 induced autophagy in macrophages and functioned as an immune adjuvant in the induction of Ag-specific immune responses. Although some of the underlying mechanisms of CCL-34-mediated T cell activation and SPM/LPM regulation were not fully investigated *in vivo*, our results revealed that autophagy was involved in CCL-34-mediated Ag processing and humoral immune responses, providing the potential application of CCL-34 as a vaccine adjuvant. These findings also highlight the potential clinical application of TLR agonist and/or autophagy-inducing agents as vaccines or adjuvants.

## Methods

### Cell lines and culture medium

RAW264.7 cells was obtained from the Bioresource Collection and Research Center and maintained in Dulbecco’s modified Eagle’s medium (Thermo Fisher Scientific, Waltham, MA, USA) containing 10% heat-inactivated bovine calf serum (Sigma-Aldrich, SIAL, USA) with 100 units/ml penicillin, 100 μg/mL streptomycin, and 0.3 mg/mL of L-glutamine (PSG, Thermo Fisher Scientific) and 1 mM sodium pyruvate (Thermo Fisher Scientific) at 37 °C in 5% CO_2_ incubator. BMDMs were cultured in RPMI medium 1640 (Thermo Fisher Scientific) supplemented with PSG, non-essential amino acids (NEAA, Thermo Fisher Scientific), 10% fetal bovine serum (Thermo Fisher Scientific), and 20 ng/mL M-CSF (R&D Systems, MN, USA). CD4^+^ T cells and stimulated BMDMs were cultured in RPMI Medium 1640 supplemented with PSG, NEAA, 10% fetal bovine serum, and mIL-2 (20 ng/mL, Peprotech, Rocky Hill, USA). For detection of IL-2, mIL-2 was removed from the complete medium.

### Antibodies and reagents

The primary antibodies used for immunoblotting were anti-LC3 antibody (Cell Signaling Technology 4108), anti-GAPDH antibody (Millipore MAB374), anti- NRF2 antibody (GeneTex GTX103322), anti-SQSTM1 antibody (GeneTex GTX100685), and anti-Beclin 1 antibody (Cell Signaling Technology 3495). The secondary antibodies used for immunoblotting were anti-mouse IgG (whole molecule)-peroxidase antibody produced in rabbit (Sigma-Aldrich A9044) and anti-rabbit IgG (whole molecule)-peroxidase antibody produced in goat (Sigma-Aldrich A0545). The pEGFP-LC3 plasmid was a gift from Dr. Tamotsu Yoshimori, Department of Genetics, Graduate School of Medicine, Osaka University. LPS-EB (LPS from E. coli O111:B4) was purchased from InvivoGen (CA, USA). 3-methyladenine and chloroquine diphosphate salt were from Sigma‐Aldrich. Bay 11–7082 was from Calbiochem (Sigma-Aldrich).

### Mice

The OT-II mice were purchased from the Jackson Laboratory, and the C56BL/6, C3H/HeN and C3H/HeJ mice were provided by National Laboratory Animal Center (NLAC), NARLabs, Taiwan. The protocols were approved by the Institutional Animal Care and Use Committee at the National Yang Ming University (IACUC Approval No: 1021236 and 1051219). All animal experiments were carried out following the National Institutes of Health Guidelines for the Care and Use of Laboratory Animals (NIH Publications No. 8023, revised 1978).

### Transfection and Establishment of Stable Cell Lines

RAW264.7 cells were transfected with pEGFP-LC3 using TurboFect (Thermo Fisher Scientific) according to the manufacturer’s instructions. The fluorescent cells were sorted by a FACSAria III cell sorter (BD Biosciences, NJ, USA) and maintained in the presence of 600 μg/mL G418 (Sigma-Aldrich).

### Immunoblotting

Cells were lysed with modified RIPA buffer (90 mM Tris, 150 mM NaCl, 1% NP40, 0.25% sodium deoxycholate, 5 mM EDTA, and 1 mM EGTA) containing protease inhibitor cocktails (Sigma‐Aldrich). The protein quantity was determined by Bradford assay (Sigma-Aldrich). Total protein (50 μg for RAW 264.7 cells or 25 μg for BMDM) was analyzed on SDS-PAGE gel and transferred onto PVDF membranes (Bio-Rad). The membranes were blocked with 5% non-fat milk or 5% BSA (Sigma‐Aldrich), incubated with the indicated primary antibodies at 4 °C overnight, and then incubated with appropriate peroxidase-conjugated secondary antibodies. The membranes were reacted with an enhanced chemiluminescence reagent (Merck Millipore, MA, USA) and the signals were detected using a Luminescence/Fluorescence Imaging System (Fujifilm, Tokyo, Japan).

### Fluorescence microscopy

Cells were grown on glass coverslips and stimulated with the candidate drugs. Cells were fixed with 4% formaldehyde, stained with the blue nuclear chromatin stain 4′, 6-diamidino-2-phenylindole, dihydrochloride (DAPI) (Sigma‐Aldrich) and mounted using ProLong Diamond Antifade Mountant (Thermo Fisher Scientific). The samples were pictured using an Olympus BX61 Microscope (Olympus Corporation, Tokyo, Japan). The percentage of cells with LC3 punctate dots was calculated to quantitate autophagy. A minimum of 150 cells per sample were counted under different experimental conditions.

### MTT cell proliferation/viability assay

RAW264.7 cells (10^4^) were cultured in 96-well plates and treated with the candidate drugs for 24 hours. Then the cells were incubated with 0.5 mg/mL MTT reagent (Sigma‐Aldrich) for 4 hours, followed by incubation with solubilization buffer (12% SDS in 45% DMF, pH=4.7). The absorbance at 550 nm (reference 650 nm) was measured by ELISA reader (Bio-Rad, Hercules, USA).

### Differentiation of murine bone marrow-derived macrophages

The bone marrow cells were isolated from C56BL/6, C3H/HeN and C3H/HeJ mice, and differentiated as described previously^64^. In brief, bone marrow cells (10^7^ cells) were cultured in complete medium. On day 2 and day 4, new medium supplemented with M-CSF (20 ng/mL, R&D Systems) was added. On day 6, non-adherent cells and loosely adherent cells were gently washed with PBS. The adherent cells were detached using 2.5 mM EDTA and reseeded for the following experiments.

### RNA extraction and the quantitative reverse transcription polymerase chain reaction (qRT-PCR)

Total RNA was extracted using TRIzol reagent according to the manufacturer’s instructions (Thermo Fisher Scientific), and the total RNA was reverse transcribed using the ThermoScript RT-PCR system (Thermo Fisher Scientific). The cDNA product was analyzed by qRT-PCR using Fast SYBR Green Master Mix (Thermo Fisher Scientific). The primer sequences were as follows. Mouse p62: ACAGCCAGAGGAACAGAT (sense) and ACAAGAATGCCAAGACACT (antisense). Mouse Il12a: TATCTCTATGGTCAGCGTTCC (sense) and TGGTCTTCAGCAGGTTTCG (antisense). Mouse Il12b: TCATCAGGGACATCATCAAACC (sense) and TGAGGGAGAAGTAGGAATGGG (antisense). Mouse GAPDH: 5′-TGTGATGGGTGTGAACCACGAG (sense) and TGCTGTTGAAGTAGCAGGAGAC (antisense). All assays were performed in triplicate using the Applied Biosystems Model 7000 instrument (Thermo Fisher Scientific). The data are quantitated using 2^−ΔCt^ (ΔCt = CtTarget gene-CtGAPDH; Ct: cycle number when the fluorescent value of the sample is equal to the threshold value).

### Measurement of Ag processing in vitro

BMDMs were stimulated with the candidate drugs in combination with 5 μg DQ-OVA (Thermo Fisher Scientific) for 24 hours. The cells were stained with anti-F4/80-PECy7 (BioLegend 123113), and analyzed by FACSCanto (BD Biosciences).

### Ag-specific T cell activation assay

BMDMs isolated from C56BL/6 mice were stimulated with the candidate drugs in the presence of 5 μM OVA-peptide 323–339 (AnaSpec, CA, USA) for 24 hours. The CD4^+^ T cells were isolated from the spleen and mesenteric lymph nodes (MLN) of OT-II mice using CD4 (L3T4) MicroBeads (Miltenyi Biotec 130–049–201), and the CD4^+^ T cells were stained with 2 μM CellTrace CFSE Cell Proliferation Kit (Thermo Fisher Scientific) for 5 minutes at 37 °C. The stimulated BMDMs (2.5×10^4^) and CD4^+^ CFSE labeled OT-II T cells (5×10^4^) were resuspended and cocultured in 96-well U bottom plates for 3 days and 5 days, respectively. The supernatant was collected on day 3 for IL-2 detection. On day 5, the supernatants were collected for IFN-γ detection, and the proliferating T cells were stained with anti-F4/80. Dead cells were excluded by staining with propidium iodide (Sigma‐Aldrich). The stained cells were acquired using FACSCanto (BD Biosciences) and analyzed by FlowJo (FlowJo, LLC, BD Biosciences).

### Cytokine ELISAs

All cytokine concentrations were measured using ELISA kits. The supernatant was collected and then detected by an IL-2 ELISA kit (R &D Systems), IL-12p70-ELISA kit (R&D systems) or an IFN-γ ELISA kit (R&D Systems) according to the manufacturer’s instructions.

### Tracing Ag processing cells in vivo

Eight- to 10-week-old C57BL/6 mice were immunized by intraperitoneal (i.p.) injection of DQ-OVA (1 mg/kg, Thermo Fisher Scientific) plus 10% DMSO or DQ-OVA (1 mg/kg) plus CCL-34 (4 mg/kg) in PBS^65^. Twenty-four hours after injection, cells from the peritoneal cavity, spleen, MLN and other lymph nodes were isolated, and the DQ-OVA^+^ cells were acquired on FACSCanto (BD Biosciences) and analyzed by FlowJo (FlowJo, LLC).

### Mouse immunizations

Eight- to 10-week-old C57BL/6 or C3H/HeN mice were immunized i.p. with PBS alone, ovalbumin (100 μg/mouse, Sigma‐Aldrich) in PBS, OVA (100 μg/mouse) plus Al(OH)_3_ (80 mg/kg, Thermo Fisher Scientific), OVA (100 μg/mouse) plus CCL-34 (4 mg/kg), or OVA (100 μg/mice) plus vehicle (10% DMSO) in a volume of 100 μl. The method and dose of OVA immunization were chosen according to a previous publication^66–68^. For the short-term immunization, the mice were sacrificed 24 hours after injection. For the long-term immunization, the mice were injected with the above-indicated drugs on day 0, day 7 and day 14, and sacrificed on day 21. For the combined treatment with autophagy inhibitor, 3-MA (20 mg/kg) was administered intraperitoneally 30 minutes before immunization as previously described^69^. The i.p. injections were carried out in awake mice without anaesthesia procedure.

### Isolation of mouse peritoneal cavity cells (PECs) and flow cytometry analysis

The PECs were collected from peritoneum, and red blood cells were removed with red blood cell lysis buffer (BioLegend 420301). The PECs were blocked with anti-CD16/32 antibody (BioLegend 101319) to exclude nonspecific binding to Fcγ receptors before surface staining. To detect dendritic cells, macrophages, neutrophils, monocytes, T cells and B cells, PECs were stained with anti-CD11c (BD Biosciences 553801), anti-mouse I-A[b] (BD Biosciences 553551 or 553552), anti-CD11b (BioLegend 101205), anti-Ly6C (BioLegend 128025), anti-F4/80 (BioLegend 123115 or 123107 or 123113), anti-CD45 (BioLegend 103115), anti-CD3 (BioLegend 100203) and anti-B220 (BioLegend 103226). To examine the activation markers, PECs or activated BMDMs were stained with anti-CD40 (BioLegend 124621), anti-CD80 (BD Biosciences 553769 or 104713), and anti-CD86 (BD Biosciences 553692 or BioLegend 105029). Cells were acquired using FACSCanto (BD Biosciences) and analyzed by FlowJo (FlowJo, LLC).

### Measurement of serum OVA-specific IgG

The levels of OVA-specific IgG in serum were measured using ELISA as described previously^70^. In brief, OVA-specific IgG was bound to an ELISA plate (Nunc, Roskilde, Denmark) coated with 2 mg/mL OVA and detected by enzyme reaction after incubating with polyclonal HRP-conjugated anti-mouse IgG (Sigma-Aldrich A9044) and tetramethylbenzidine. After the enzyme reaction, absorbance was measured at 450 nm (reference 540 nm).

### Data and statistical analyses

All the data from at least three independent experiments were analyzed with Student’s t-test by GraphPad Prism version 8.1.0 for Windows (GraphPad Software Inc., La Jolla, USA, www.graphpad.com). All the results are shown as the mean ± SD, and statistical significance is indicated by * (*p* < *0.05*) for comparison between untreated and treated groups.

## Supplementary information

Supplementary Information.
