# Supplementary material for: Vaccine adjuvant activity of a TLR4-activating synthetic glycolipid by promoting autophagy
Source: Sci Rep. 2020 May 21;10:8422. doi: 10.1038/s41598-020-65422-1 (PMC7242473; doi:10.1038/s41598-020-65422-1)
Supplement: Supplementary file 1 — Supplementary Information. [file 41598_2020_65422_MOESM1_ESM.pdf]

Supplementary information

Vaccine adjuvant activity of a TLR4-activating synthetic glycolipid by promoting autophagy

Yi-Ju Chou[1], Ching-Cheng Lin[2], Ivan Dzhagalov[2], Nien-Jung Chen[2], Chao-Hsiung Lin[3], Chun-Cheng Lin[4], Szu-Ting Chen[5], Kuo-Hsin Chen[6]*and Shu-Ling Fu[1, 7]*

[1]Program in Molecular Medicine, School of Life Sciences, National Yang-Ming University and Academia Sinica, Taipei 11221, Taiwan
[2]Institute of Microbiology and Immunology, National Yang-Ming University, Taipei 11221, Taiwan;
[3]Department of Life Sciences and Institute of Genome Sciences, National Yang-Ming University, Taipei 11221, Taiwan
[4]Department of Chemistry, National Tsing Hua University, Hsinchu 300, Taiwan
[5]Institute of Clinical Medicine, National Yang-Ming University, Taipei 11221, Taiwan
[6]Department of Surgery, Far-Eastern Memorial Hospital, New Taipei City 22060, Taiwan
[7]Institute of Traditional Medicine, National Yang-Ming University, Taipei 11221, Taiwan

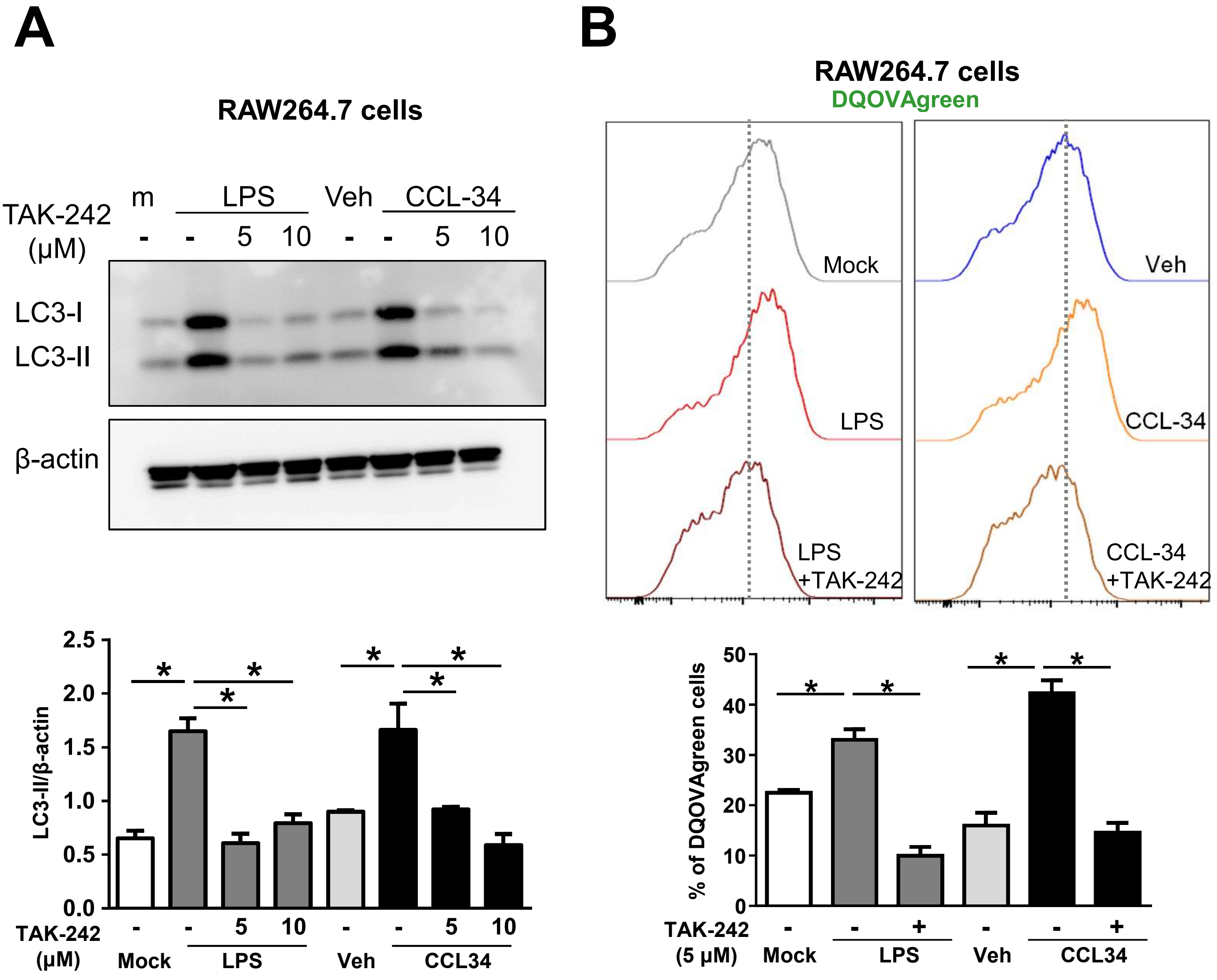

Fig. S1 CCL-34 enhanced autophagy and antigen processing in RAW264.7 cells in a TLR4-dependent manner

RAW264.7 cells (2.5 x 10^5) were pretreated with TAK-242 (5 μM and 10 μM, ApexBio Technology, Boston, MA) for 2 hours and then incubated with LPS (100 ng/mL), vehicle (0.1% DMSO), or CCL-34 (30 μM) for 24 hr. **(A)** The LC3-II protein was detected by immunoblotting, using β-actin as an internal control (n=3).**(B)** RAW264.7 cells (2.5 x 10^5) were incubated with candidate drugs in combination with 5 μg DQ-OVA for 24 hours. A representative histogram showing the flowcytometric analysis of DQ-OVAgreen$^+$ RAW264.7 cells and the quantitative data are shown (n=5).

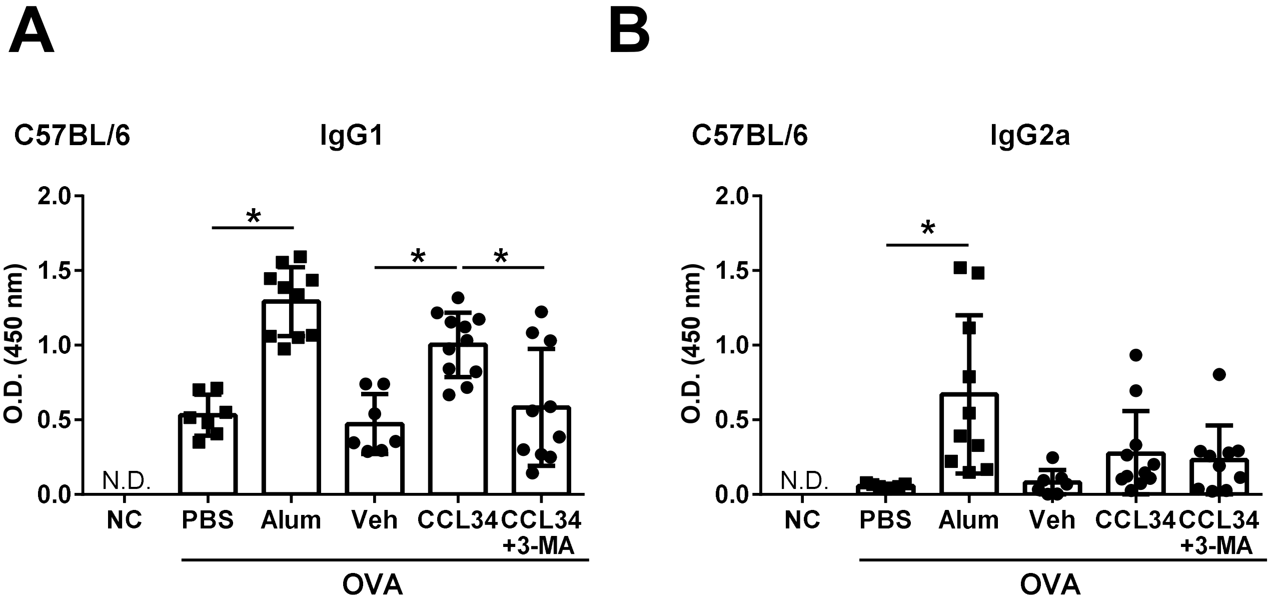

Fig. S2 CCL-34 induces OVA-specific IgG1 and IgG2a productions in CCL-34/OVA-immunized

Mice were immunized with OVA (100 μg/mice) alone or OVA formulated with Alum (80 mg/kg), vehicle (10% DMSO) or CCL-34 (4 mg/kg) on days 0, 7 and 14. The serum was collected on day 21 and analyzed OVA-specific IgG1 **(A)** and IgG2a **(B)** using ELISA (the number of mice was PBS=5, PBS plus OVA=7, Alum plus OVA=5, vehicle plus OVA=7, CCL-34 plus OVA=11, and CCL-34, 3-MA plus OVA=10 for total two trials).

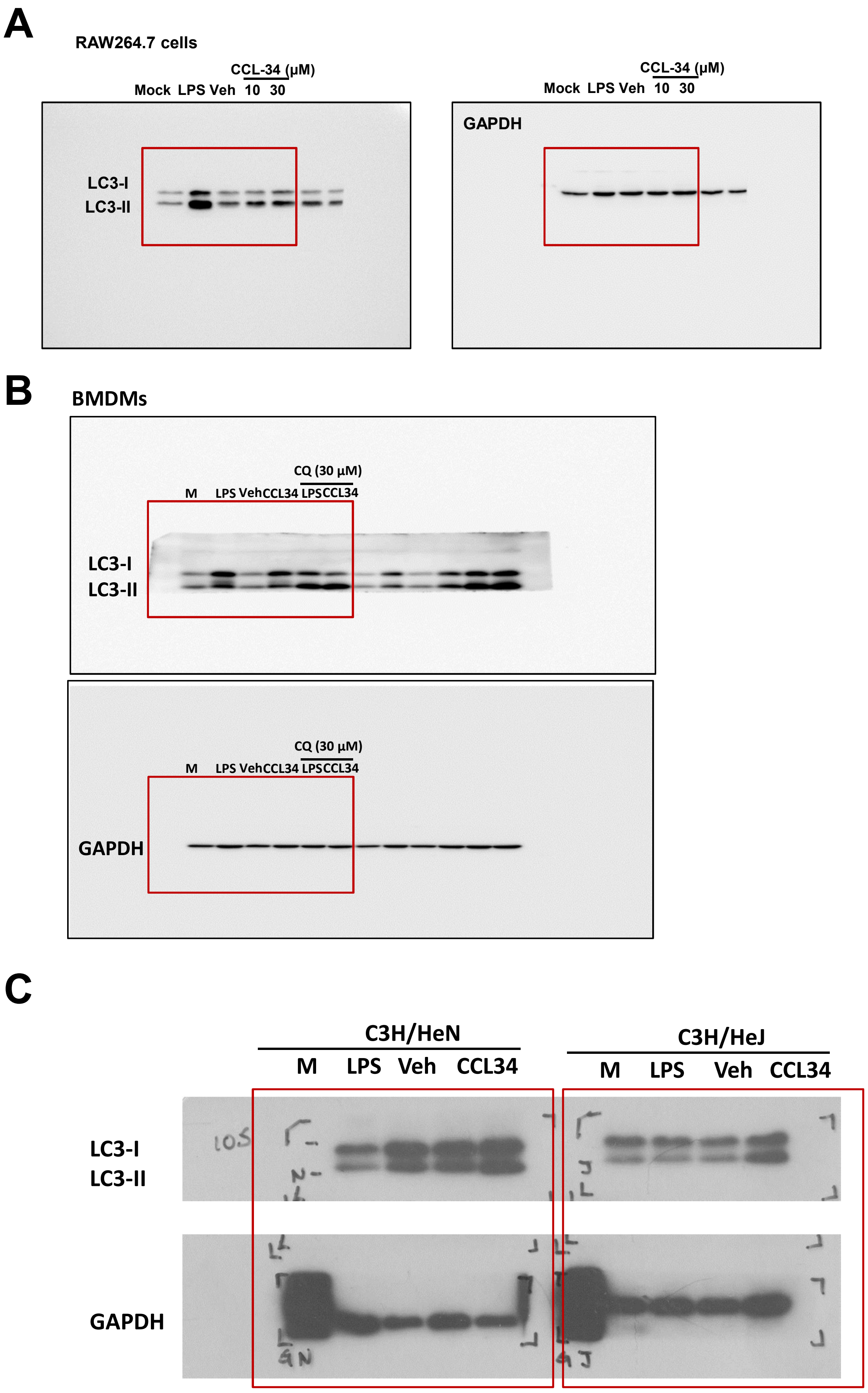

Fig. S3 The full-length blots of Fig. 1B (A), Fig. 1F (B) and Fig. 1G (C)

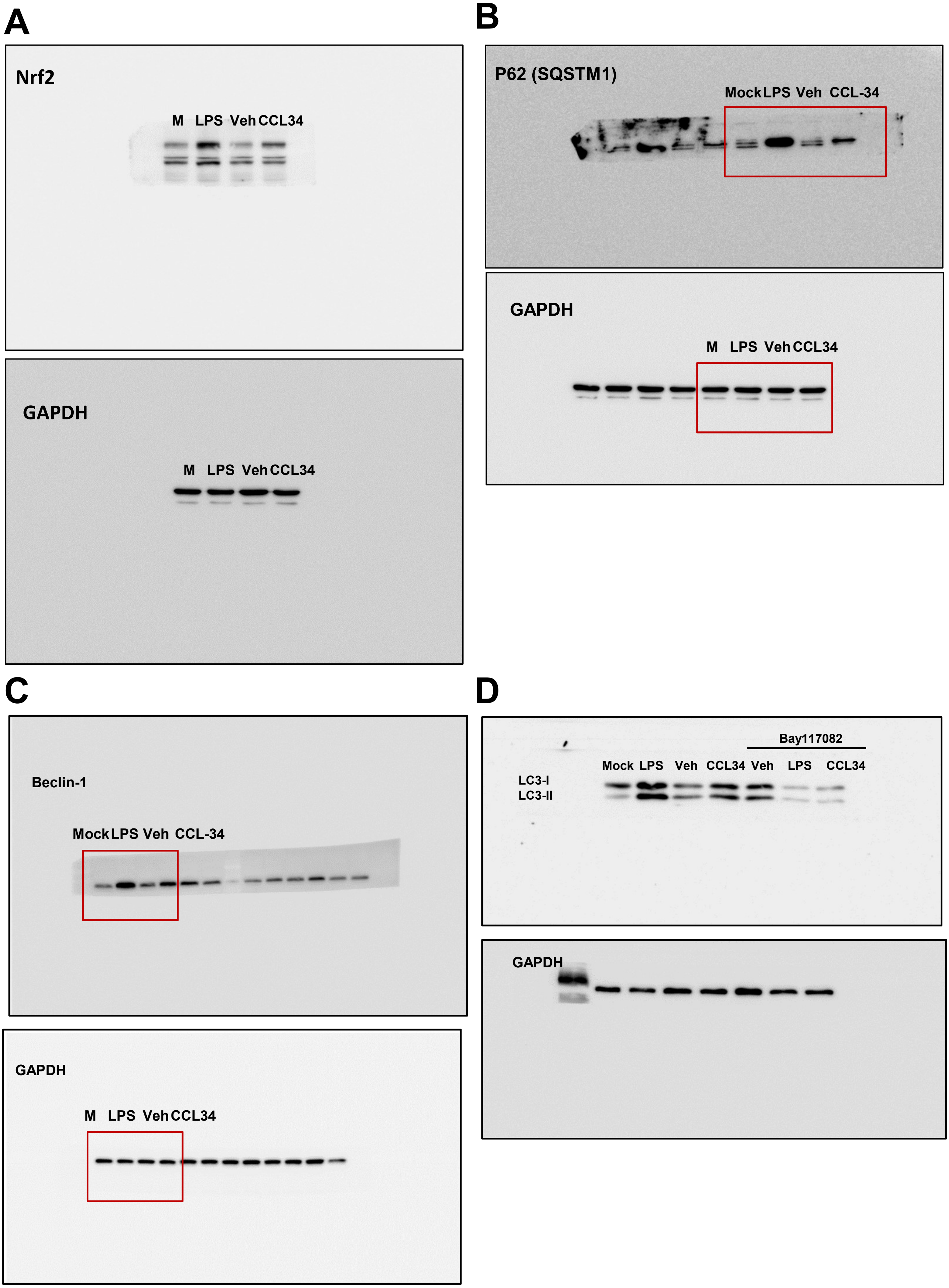

Fig. S4 The full-length blots of Fig. 2A (A), Fig. 2C (B), Fig. 2D (C) and Fig. 2E (D)

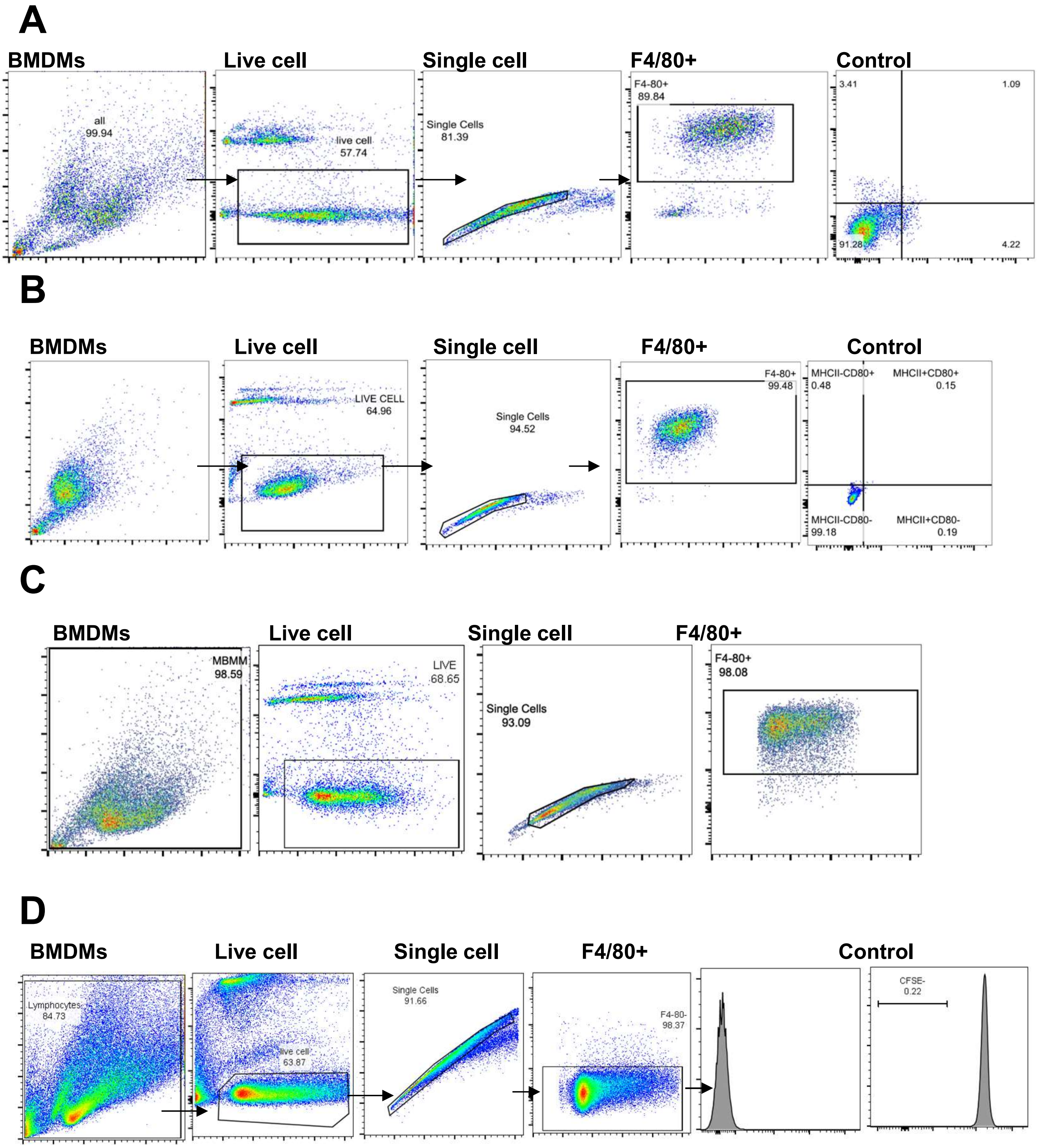

Fig. S5 The gating strategy and control of Fig. 3A (A), Fig. 3B (B), Fig. 3C (C) and Fig. 3D (D)

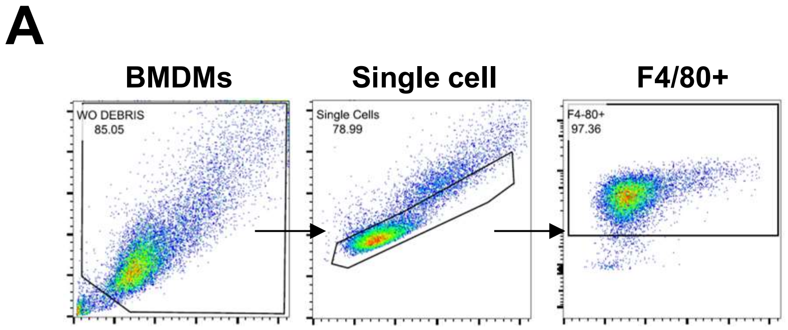

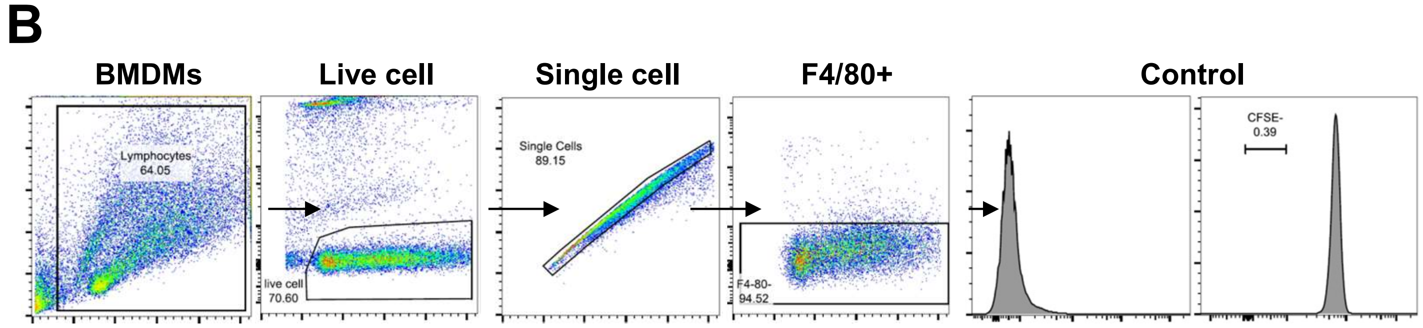

Fig. S6 The gating strategy and control of Fig. 4A (A) and Fig. 4B (B)

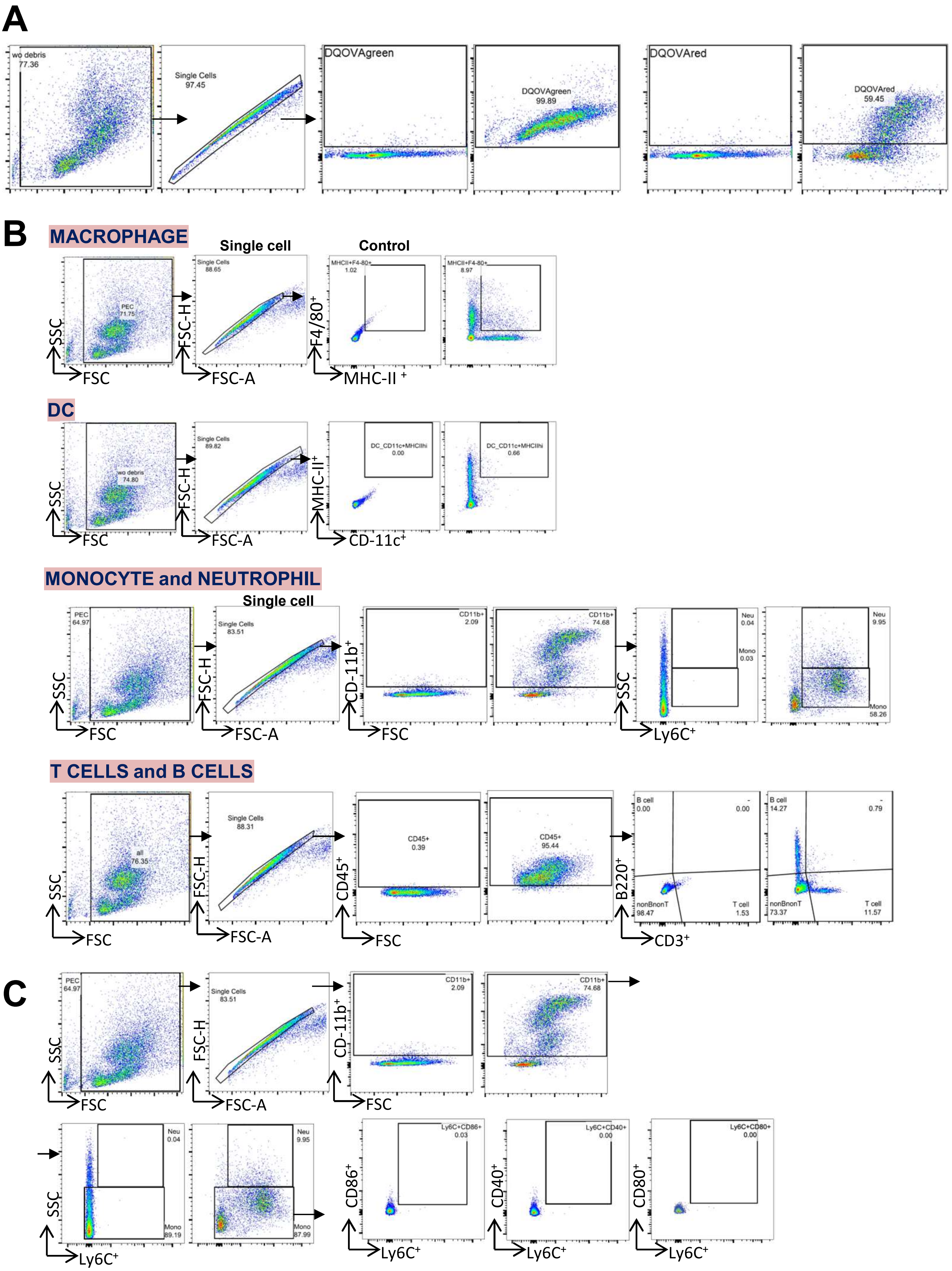

Fig. S7 The gating strategy and control of Fig. 5A (A), Fig. 5C (B) and Fig. 5D (C)
